# Source-aware microbial–host signaling after ischemic stroke: evidence, competing origins, and causal roles

**DOI:** 10.3389/fimmu.2026.1986786

**Published:** 2026-10-19

**Authors:** Xinwei Zhao, Wanyi Zhang, Zeyu Yang, Zhengyuan Liu, Linlin Zhang, Zelin Chen

**Affiliations:** 1 Tianjin University of Traditional Chinese Medicine, Tianjin, China; 2 Shanxi University of Traditional Chinese Medicine, Jinzhong, Shanxi, China; 3 Shandong University of Traditional Chinese Medicine, Jinan, Shandong, China; 4 Encephalopathy Acupuncture Center 2, The Second Affiliated Hospital of Tianjin University of Traditional Chinese Medicine, Tianjin, China

**Keywords:** DAMPs, gut microbiota, intestinal barrier, ischemic stroke, microbial metabolites, microbial translocation, neuroinflammation, neurovascular unit

## Abstract

Ischemic stroke initiates a well-established sterile inflammatory response in injured brain tissue. Experimental studies report post-stroke intestinal barrier and mucosal changes, viable bacterial dissemination to peripheral compartments in selected animal models, and microbiota-associated material in ischemic brain tissue. Human cohorts associate gut community or metabolite profiles with infection and clinical outcomes, while microbiome-based models predict stroke-associated pneumonia or functional outcomes. These observations differ in signal identity, sampling time, source tracing, and measured destination. Gut-to-periphery dissemination in one model does not establish that brain-associated signals are viable or intestinal in origin; oral, airway, aspiration-related, infectious, and other peripheral sources remain plausible. This narrative Review synthesizes human and experimental evidence across five connected links: anatomical source, molecular identity, transport route, receptor or sensing cell, and tissue consequence. We distinguish transport from recognition and source attribution from causal-role assignment. Current evidence supports context-dependent intestinal participation and established brain-derived damage-associated molecular pattern (DAMP) signaling, but not a universal gut-to-brain causal cascade. We outline longitudinal source-to-target sampling, barrier and exposure measurements, signal-specific tracing, and timed perturbation to test each link.

## Graphical Abstract

**Figure:**
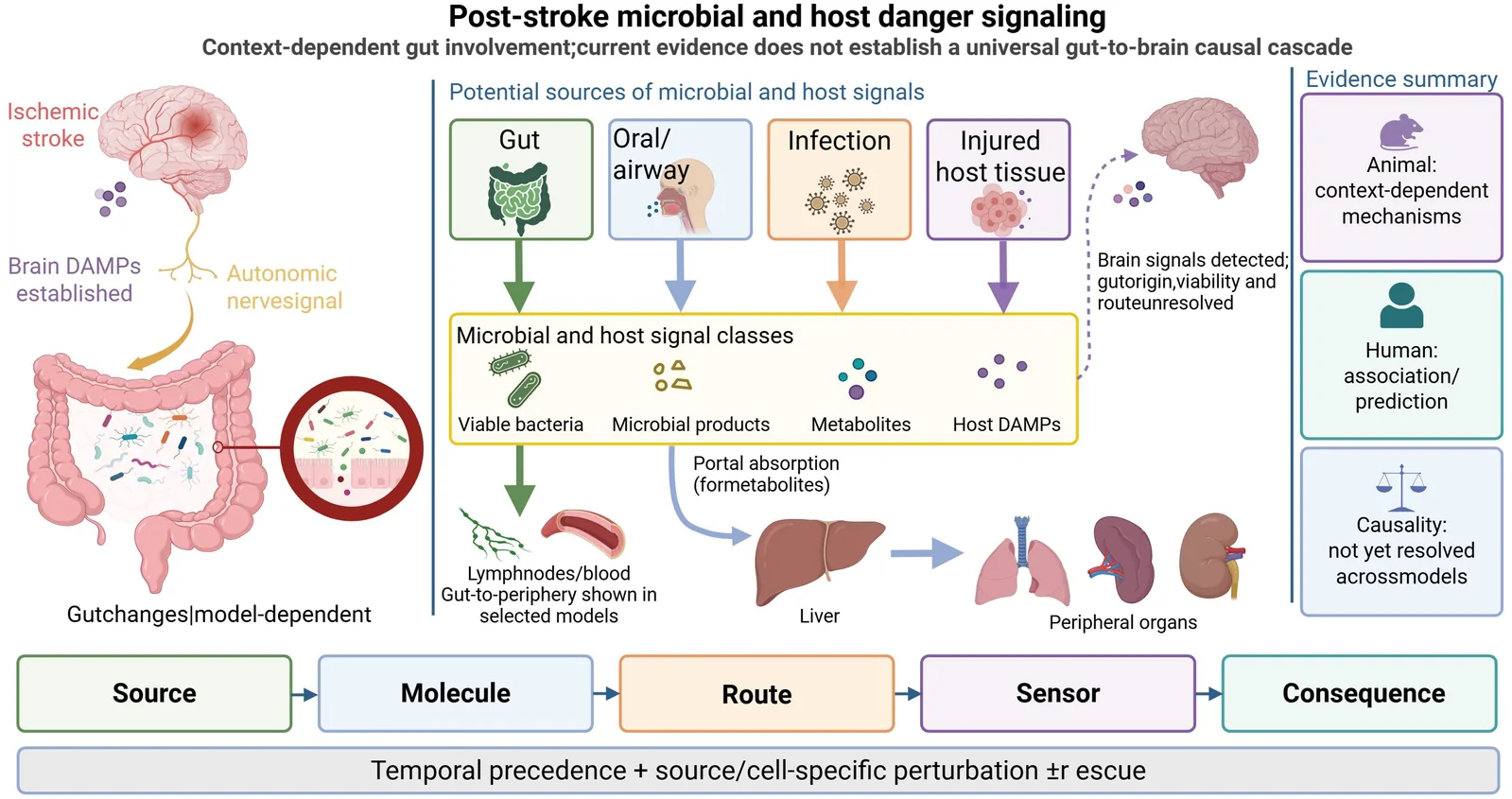

## 1 Evidence base and the source-attribution framework

### 1.1 Established sterile inflammatory signaling after ischemia

Ischemic stroke initiates a well-characterized sterile inflammatory response in injured brain tissue (1, 2). Experimental studies implicate peroxiredoxins, high-mobility group box 1 (HMGB1), mitochondrial DNA (mtDNA), adenosine triphosphate (ATP), and oxidized lipids in post-ischemic signaling through Toll-like receptors (TLRs), the receptor for advanced glycation end products (RAGE), cyclic GMP–AMP synthase (cGAS)–stimulator of interferon genes (STING), and NLR family pyrin domain containing 3 (NLRP3) pathways (1–12). In parallel, stroke studies report changes in enteric neuronal, epithelial, barrier, and mucosal function (13–17), and have recovered viable bacteria from mesenteric lymph nodes, blood, lung, and other peripheral tissues in selected models (18–22). Together, these studies establish brain-derived sterile inflammation, post-stroke intestinal changes, and bacterial dissemination to peripheral compartments as distinct findings.

The experimental findings differ in the links they establish. Stanley et al. linked a small-intestinal source to a post-stroke lung community (20), whereas an exploratory transient middle cerebral artery occlusion (MCAO) study found no consistent functional permeability or bacterial translocation during the first 72 h (23). Peh et al. detected microbiota-dependent bacterial-associated material in ischemic brain tissue (24), a finding distinct from source-traced gut-to-periphery dissemination. Together, these studies establish different links and motivate separate evaluation of source, route, and tissue consequence.

### 1.2 Why anatomical source remains unresolved

Human cohorts report intestinal injury markers and time-dependent circulating lipopolysaccharide (LPS) (25–27), changes in gut and oral microbial communities or metabolite profiles (28–53), and associations between microbiome features and infection, cognition, rehabilitation, prediction, or functional outcome (25–27, 36, 37, 40–56). These studies establish clinical associations, systemic exposure, and predictive signals. Oral–gut overlap and variation in infection, aspiration, clearance, medication, and sampling conditions mean that these observations alone do not assign an individual extra-intestinal signal to the gut (25–27, 38, 39).

This Review synthesizes human and experimental evidence to determine when a microbial signal detected outside the intestine can be attributed to the gut, which measured routes support its movement, and whether the exposure contributes to a defined post-stroke consequence. Section 1.3 introduces the framework used to organize source, molecular identity, transport, sensing, and tissue consequence, while source attribution and causal-role assignment are evaluated separately.

### 1.3 Source framework and inference rules

We organize the evidence using a five-link framework: (i) anatomical source, such as the gut, brain, oral cavity, airway, an infected site, or another peripheral tissue; (ii) molecular identity, such as LPS, peptidoglycan, bacterial DNA, mtDNA, or a chemically defined metabolite; (iii) transport route, including passage across epithelial barriers into blood or lymph, physiological intestinal absorption into portal blood, aspiration, or circulation from a local release site; (iv) receptor or sensing cell; and (v) tissue consequence. We assess these links separately because evidence establishing one does not, by itself, establish the others.

In this Review, we reserve “source-resolved” for cases in which source-discriminating evidence links a signal detected in a target compartment to a defined anatomical source. Examples include molecular- or strain-level tracing from a defined source or source-restricted colonization followed by matched recovery in the target (20). Selective manipulation of a source that changes the target signal supports source contribution, but without source-discriminating linkage it is not, by itself, source-resolved. Detection of the same taxon or molecule in paired source and target samples supports concordance but, without source-discriminating linkage, does not establish transfer direction (20, 24). A germ-free comparison combined with tissue localization supports microbiota dependence but does not, by itself, identify the intestine as the anatomical source (24). Findings compatible with an intestinal contribution but lacking source-discriminating linkage are described as “source-aware,” and the inference they support is stated explicitly (20, 24). Source attribution asks where a signal originates; route inference asks how it reaches the target; causal attribution asks whether the exposure changes a defined tissue consequence and therefore requires outcome-directed evidence (20, 24, 57, 58).

### 1.4 Scope, search strategy, and evidence appraisal

This is a focused narrative Review rather than a systematic review or meta-analysis. PubMed/MEDLINE was searched from database inception through 2 September 2026 using six source-aware concept blocks covering: (i) ischemic stroke or cerebral ischemia; (ii) gut microbiota, oral microbiota, or microbiome; (iii) intestinal barrier, permeability, or translocation; (iv) LPS, peptidoglycan, bacterial DNA, short-chain fatty acids (SCFAs), acetate, indoles, or trimethylamine N-oxide (TMAO); (v) damage-associated molecular patterns (DAMPs), HMGB1, mtDNA, Toll-like receptors 2/4/5, NLRP3, STING, triggering receptors expressed on myeloid cells 1/2 (TREM1/TREM2), or the gamma-aminobutyric acid type A receptor (GABAA); and (vi) infection, aspiration, pneumonia, or the neurovascular unit. Database searching was supplemented by backward citation screening of key reviews and primary studies, with particular attention to source tracing, intestinal barrier function, infection, transport routes, receptor mechanisms, and recent human cohorts. The search is reported at the concept-block level to define the scope of this narrative synthesis; it is not presented as an exhaustive or reproducible systematic search.

Eligible evidence included peer-reviewed human, animal, and mechanistic studies directly addressing post-stroke microbial ecology, intestinal injury, microbial products or metabolites, host DAMPs, or innate-immune relays. Non-stroke studies were retained only when they clarified receptor, transport, or metabolite biology relevant to interpretation and are treated as indirect biological evidence (11, 59–65). Duplicate reports, conference-only records, and studies without source-, route-, signal-, or mechanistically relevant endpoints were excluded during curation. Because this was a narrative synthesis, no Preferred Reporting Items for Systematic Reviews and Meta-Analyses (PRISMA) flow diagram, preregistered protocol, pooled effect estimate, or formal risk-of-bias instrument was used.

For each included study, we recorded the population or experimental species, stroke model and sampling time, signal or endpoint, anatomical compartment, source-tracing or perturbation approach, outcome, and key limitation. Evidence is described according to the question directly addressed—for example, association, extra-intestinal exposure, route or source tracing, receptor or sensing-cell engagement, and perturbational evidence of biological or causal relevance. These categories are not treated as a single evidence ladder. Human associations, stroke-specific experimental mechanisms, and indirect biological evidence are interpreted separately, and no formal ranking of LPS or other candidate signals is attempted because inferential strength depends on assay performance, timing, contamination and infection control, and source-attribution quality.

## 2 From autonomic intestinal injury to microbial exposure

### 2.1 Barrier and mucosal checkpoints

Post-stroke intestinal injury is a dynamic and conditional phenotype rather than a universal event. Autonomic stress, local hypoperfusion, reduced intake, altered transit, epithelial cell death, and changes in mucosal immunity can affect tight junctions, mucus architecture, antimicrobial programs, immunoglobulin responses, and motility. Permanent MCAO and severe experimental stroke have been associated with early ileal injury and culture-positive dissemination, whereas immobilization stress can intensify colonic inflammation and bacterial translocation (18, 19). In mice, increased permeability preceded recovery of commensals from peripheral organs, and paired sampling with oral inoculation identified the small intestine as the source of a post-stroke lung community in a selected model (20). Aged mice show impaired clearance of extra-intestinal bacteria and greater susceptibility to gut-associated infection, linking host age to both duration and consequence of exposure (21, 22).

Timing materially changes the interpretation. In a permanent MCAO study, intestinal permeability and epithelial death increased within hours, sympathetic denervation reduced the early phenotype, and barrier function largely recovered by 24 h (14). Stroke also alters enteric neuronal function, smooth-muscle relaxation, and transit, changes that can be attenuated by chemical sympathectomy (13). In contrast, an exploratory transient MCAO study found reduced ileal claudin-1 without a clear increase in functional sugar-probe permeability or consistent bacterial translocation during the first 72 h (23). Longitudinal experimental studies further show that mucosal and community abnormalities may persist or evolve during recovery even when an early permeability phenotype has changed (15, 16). Taken together, lesion model, reperfusion, age, sex, assay choice, and sampling time determine whether a leaky-gut phenotype is observed.

Mucosal immune surveillance may fail before gross permeability becomes measurable. Stroke-induced sympathetic activity can downregulate epithelial TLR5, weaken flagellin-specific immunoglobulin A (IgA) and antimicrobial defense, and permit mucosal penetration by commensal organisms in experimental models; patient-linked noradrenaline and flagellin measurements support clinical relevance, although organismal dissemination was not measured (17). Noradrenergic signaling can also induce triggering receptor expressed on myeloid cells 1 (TREM1) in inflammatory lamina-propria macrophages, creating a feed-forward loop that weakens mucosal defense and amplifies injury (66). Age-related microbiota changes and matrix metalloproteinase 7 (MMP-7)-related barrier biology add further context (67, 68). These data support multiple mucosal checkpoints rather than a single universal permeability switch.

Compartment-specific receptor manipulation sharpens this inference. In mice, intestinal epithelial TLR4 deletion produced greater protection than brain-specific TLR4 deletion in an acute stroke model, while pharmacological activation worsened intestinal and cerebral phenotypes; intestinal GABA_A_ receptor blockade modified the protective effect, and microbiota changes involving *Bacteroides* and *Prevotella* tracked with the epithelial response (58). This study supports a gut-compartment receptor relay through compartment-specific perturbation, although the luminal ligand and transport route were not identified (58).

### 2.2 Microbial ecology and metabolites

Across clinical cohorts, ischemic stroke is associated with altered gut community composition or metabolite profiles, but reported taxa vary across populations (28–37, 40–53). Experimental studies likewise link microbiota manipulation to stroke phenotypes, while the direction and magnitude of effects vary with age, sex, diet, housing, antibiotic exposure, and model (66, 67, 69–79). Together, these studies show that stroke-associated community profiles and microbiota-related effects vary across cohorts, host backgrounds, and experimental conditions (28–37, 40–53, 66, 67, 69–79).

Microbial metabolites represent an exposure class distinct from viable bacteria and structural microbial products (60–65, 80–92). SCFAs and related metabolites have been linked to epithelial function, immune regulation, microglial responses, and recovery phenotypes in experimental stroke models (60–62, 81, 82, 86). In one experimental study, valerate worsened outcome in aged mice (80). Separately, a cohort with large-artery atherosclerotic stroke or transient ischemic attack reported lower circulating TMAO than controls (29). These findings concern different molecules, populations, and outcomes and should not be read as a direct contrast. SCFAs measured at thrombectomy tracked with inflammatory markers and discharge symptoms but were independent of initial stroke severity (35). Differences in dose, host age and metabolic status, and sampling window constrain direct comparisons across metabolite studies (29, 35, 54, 80–86, 93).

Host-context studies ask whether age or metabolic disease changes microbiota-associated phenotypes. An age-focused study linked acetate-related measures with recovery in older adults and tested young-donor microbiota transfer in aged mice (54). A separate diabetes-focused study reported reduced diversity and *Bacteroides* in diabetic stroke mice, with a similar pattern in patient samples (93). Together, these studies illustrate that age and diabetes modify microbiota- or metabolite-associated stroke phenotypes (54, 93).

Barrier- and recovery-focused cohorts address a different question. The Italian rehabilitation cohort examined longitudinal stool profiles, fecal metabolites, and fecal zonulin in relation to permeability-related measures and recovery (94). This exploratory, stool-centered cohort associated microbial measures with permeability-related markers and recovery, but did not directly measure systemic transport (94).

Post-stroke studies measure distinct endpoints, including community shifts, circulating microbial products, viable bacteria in peripheral tissues, brain-associated microbial signals, and outcome prediction (24–37, 55–57, 95). Sections 3 and 4 examine transport routes and receptor/sensing-cell relays, whereas Section 5 synthesizes human associations, sampling time, prediction, and source attribution (20, 23, 24, 55–57, 95). **Table 1** summarizes candidate sources, route options, sensing pathways, and interpretive boundaries by signal class.

**Table 1:** Signal classes, routes, recognition, and evidence boundaries.

Signal class	Candidate source(s)	Possible route	Recognition/relay	Evidence state and interpretive boundary
Host-derived DAMPs	Injured brain and other damaged host tissues	Local release; cerebrospinal fluid; blood	TLR2/4, RAGE, cGAS-STING, NLRP3	Sterile inflammatory pathways are established in experimental stroke (1–10). Shared receptor activation must not be interpreted as microbial exposure.
Viable bacteria	Gut, oral cavity, airway, or another colonized/infected site	Barrier crossing to lymph/blood; aspiration; local-to-systemic dissemination	Organism-dependent pattern-recognition receptors (PRRs) and host-cell responses	Selected models report culture-positive bacterial recovery in peripheral compartments and source-linked gut-to-periphery dissemination (18–22). Functional leak and translocation were not consistent across all models (23); gut Enterobacteriaceae expansion was not required for lung colonization in another model (95). Interpret each result within its model and use viability and source controls for live-bacteria claims.
Structural microbial products	Microbial communities or infection; anatomical source often unresolved	Barrier crossing; blood/lymph; local tissue exposure	LPS: LBP-CD14-MD-2/TLR4; other products: TLR2, NOD1/2, endosomal/cytosolic sensors	Peh et al. detected peptidoglycan/LPS-positive structures and bacterial 16S signal in blood and ischemic brain; the germ-free comparison supports microbiota dependence, but strain-level gut-to-brain origin and viability in brain remain unresolved (24). Molecular detection or receptor recognition does not by itself demonstrate intestinal crossing (11, 24, 57, 59, 96).
Microbial metabolites	Microbiota plus diet and host metabolism	Physiological absorption; portal metabolism; systemic circulation	G-protein-coupled receptors (GPCRs), histone deacetylases, metabolic and immune pathways	Context-dependent associations and experimental effects (54, 60–65, 80–86, 89, 90, 92, 97, 101–104). Do not label metabolites as bacterial translocation; account for diet, medication, kidney and liver function.
Community composition	Stool, oral, or airway microbiota	Composition alone does not measure transport	Not applicable	Human association and prediction studies (28–44, 47–53, 55, 56). Taxonomic abundance does not prove source, passage, or causality.

Distinct signal classes require different route models and evidentiary standards. DAMPs, viable microorganisms, structural microbial products, metabolites, and microbial community composition should not be grouped under a single concept of “translocation.” Source attribution and causal role are evaluated separately. NOD1/2 denotes nucleotide-binding oligomerization domain-containing proteins 1 and 2.

## 3 Route-specific evidence for microbial signals

### 3.1 Viable bacterial dissemination

Reports of viable bacteria after stroke include recovery from mesenteric lymph nodes, blood, lung, and other peripheral tissues in selected models (18–22). Findings are not uniform: Oyama et al. reported reduced ileal claudin-1 without consistent functional permeability or bacterial translocation during the first 72 h (23). Peh et al.’s brain findings are treated below as bacterial-associated material rather than viable dissemination (24). These destinations represent different anatomical endpoints and should not be treated as evidence for a single route. Lymphatic and blood-borne transport, aspiration-related exposure, and organ-specific dissemination require different route-appropriate measurements. **Figure 1** maps these distinctions by signal class and separates measured links from unresolved paths.

**Figure 1:**
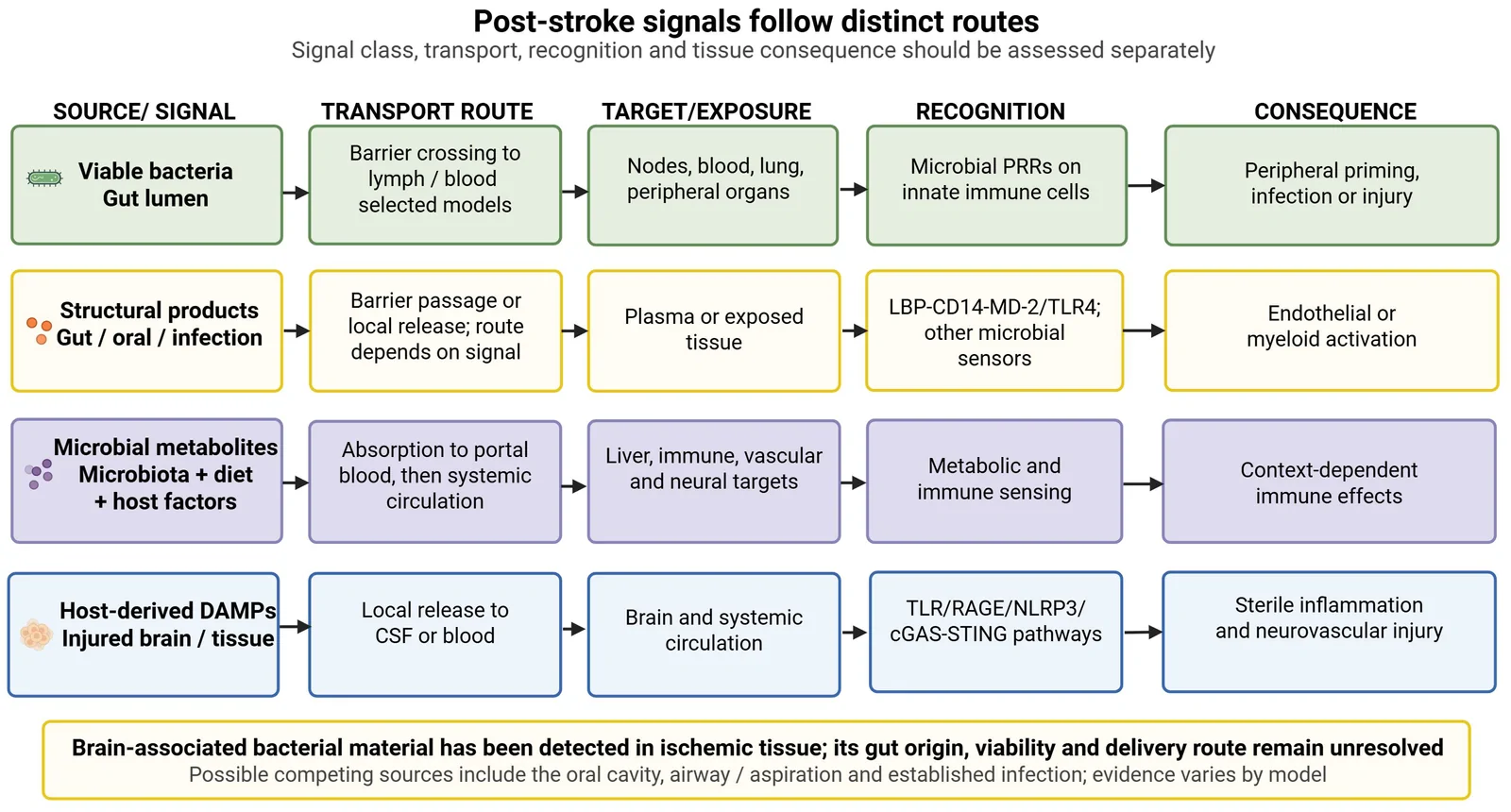
Post-stroke signals follow distinct routes. Viable bacteria, structural microbial products, metabolites, and host-derived DAMPs are shown separately because they require different transport models. The arrows organize candidate links from signal class to target exposure, recognition, and tissue consequence; they do not indicate that every route is established in every model. Brain-associated bacterial material has been reported, but its intestinal origin, viability, and delivery route remain unresolved (20, 24, 95). BBB, blood–brain barrier; cGAS, cyclic GMP–AMP synthase; CD14, cluster of differentiation 14; CSF, cerebrospinal fluid; DAMPs, damage-associated molecular patterns; LBP, lipopolysaccharide-binding protein; MD-2, myeloid differentiation factor 2; NOD1/2, nucleotide-binding oligomerization domain-containing protein 1/2; NLRP3, NLR family pyrin domain containing 3; PRRs, pattern-recognition receptors; RAGE, receptor for advanced glycation end products; STING, stimulator of interferon genes; TLR, Toll-like receptor; TREM1, triggering receptor expressed on myeloid cells 1.

Stanley et al. combined 16S ribosomal RNA (rRNA)-based source analysis, paired tissue sampling, and oral inoculation to link a small-intestinal source to a post-stroke lung community (20). In that model, increased gut permeability preceded dissemination of orally inoculated bacteria to peripheral tissues, supporting directional gut-to-periphery transport under the tested conditions (20). The study did not distinguish lymphatic from blood-borne movement or test direct brain delivery (20). In another model, suppressing the post-stroke gut Enterobacteriaceae bloom did not prevent lung colonization (95). Together, these studies show source-linked gut-to-periphery dissemination in one setting and gut-bloom-independent lung colonization in another (20, 95).

### 3.2 Structural microbial products

Structural microbial products can disseminate without viable organisms. LPS, peptidoglycan, bacterial lipoproteins, and microbial nucleic acids differ in chemical identity, assay limitations, receptor recognition, and plausible route. Their detection outside the intestine can indicate microbial exposure even when viability and anatomical origin remain unresolved.

The diabetic endotoxemia model links intestinal permeability, plasma LPS, brain-associated LPS-TLR4 signaling, and outcome (57). Separately, a germ-free comparison and tissue localization in the 2026 mouse study support microbiota dependence of at least some brain-associated bacterial signals but do not resolve intestinal origin or viability in brain (24). *Enterococcus*-derived cytolysin provides a different example: a defined microbial product can aggravate experimental ischemic injury through NLRP3-related barrier disruption (96). Structural microbial products should therefore be reported by molecule and compartment rather than grouped under a generic translocation label.

### 3.3 Metabolites are not translocation endpoints

Metabolites generated or modified by the microbiota can reach the host through normal absorption, portal circulation, hepatic metabolism, and systemic distribution. They should not be grouped with bacterial translocation. A circulating metabolite can reflect microbial production, diet, host metabolism, medication, or renal and hepatic clearance. Source attribution therefore requires targeted chemical identification, time-matched luminal, portal, and systemic measurements where feasible, and isotope or defined-colonization experiments when an intestinal origin is claimed.

Experimental stroke studies support biological effects of SCFAs, indole-3-propionic acid, indole-lactic acid, TMAO-related pathways, hydrogen-sulfide-related signaling, and other metabolites (54, 63–65, 80–86, 89–92, 97–104). These studies support metabolite-responsive mechanisms in experimental stroke but do not directly trace metabolite delivery to brain (54, 63–65, 80–86, 89–92, 97–104). Their interpretation depends on chemical identity, production and absorption, host context, dose, and target tissue.

### 3.4 Transport must be separated from recognition

The LPS transfer cascade illustrates the distinction between route and recognition. Lipopolysaccharide-binding protein (LBP) binds LPS and transfers it to cluster of differentiation 14 (CD14) for presentation to myeloid differentiation factor 2/TLR4 (MD-2/TLR4) (59). This identifies ligand presentation and receptor recognition, not epithelial crossing or delivery to a specified organ. The same principle applies to bacterial DNA, peptidoglycan fragments, and metabolites: route-specific measurements should support transport before receptor-specific perturbation is used to identify sensing (11, 57, 59).

## 4 Recognition pathways and downstream relays

### 4.1 Shared receptors integrate different upstream signals

Post-stroke innate sensors are convergence nodes. TLR2 and TLR4 have documented roles in ischemic injury (12), yet TLR4 can respond to microbial LPS and host-derived ligands such as HMGB1 (11). NLRP3 can be activated by microbial material, mtDNA, ATP, oxidative stress, ionic disturbance, and other sterile signals (5, 6, 9, 10). STING-related signaling can arise from damaged host mitochondria and can interact with NLRP3 in microglia (5, 6, 8, 105). TREM1 mainly amplifies myeloid inflammatory responses and may integrate brain- and intestine-associated immunogens (66). Because TLR4 and NLRP3 integrate microbial and host-derived inputs, their activation identifies a convergent sensing pathway rather than a unique anatomical source (5, 6, 9–12).

Brain-derived DAMP pathways remain an essential alternative and can coexist with microbial exposure. Peroxiredoxins act as post-ischemic inflammatory initiators, HMGB1 is associated with human outcome, and interferon-induced protein 35 (IFP35) has been proposed as a stroke-linked DAMP engaging TLR4–nuclear factor kappa B (NF-κB)–NLRP3 signaling (3, 4, 7). mtDNA-STING signaling and astrocytic cGAS-STING regulation provide additional brain-led mechanisms (5, 6, 105). These pathways support a dual-source model in which host and microbial signals may converge without being anatomically interchangeable.

### 4.2 Intestinal and peripheral immune relays

The intestine can influence systemic and cerebral inflammation through peripheral immune relays. The microbiota regulates intestinal gamma-delta T-cell programming and meningeal interleukin-17 (IL-17) responses in experimental stroke (70). Peripheral TREM1 responses to brain and intestinal immunogens can amplify severity through myeloid cells (66). Stroke-induced sympathetic activity can compromise epithelial survival, TLR5-mediated mucosal defense, and motility (13, 14, 17). Intestinal epithelial TLR4-GABAA signaling provides a compartment-specific receptor mechanism (58). Together, these studies support intestinal and peripheral immune relays that can modify cerebral responses (13, 14, 17, 58, 66, 70).

Adaptive and border-tissue immune cells further complicate simple gut-to-brain narratives. Single-cell and flow-cytometry work has identified distinct post-stroke states of dural and brain-infiltrating IL-17-producing gamma-delta T cells (106). Such data emphasize that anatomical location and cell state matter when a shared cytokine pathway is interpreted. Vagal signaling and mast-cell regulation offer another route by which intestinal state can modify barrier and neurovascular phenotypes (87).

### 4.3 The neurovascular unit as a convergence site

The neurovascular unit integrates endothelial activation, leukocyte trafficking, coagulation, microglial and astrocytic responses, and blood–brain-barrier injury. Circulating microbial products or metabolites can prime peripheral myeloid and endothelial cells, while brain-derived DAMPs can initiate local responses; both streams can increase cytokines, proteases, extracellular traps, reactive oxygen species, and barrier dysfunction (1, 2, 9, 10, 30, 31, 33, 66, 96). The neurovascular unit therefore provides a site where local DAMP-driven responses and circulating microbial or metabolic signals may converge.

A two-compartment model is more consistent with the current evidence than a universal one-way gut-to-brain cascade. Brain injury can initiate sterile inflammation, while intestinal changes can alter the peripheral immune and metabolic environment. In selected models, bacterial products or signals may subsequently be detected in ischemic tissue when systemic exposure and barrier dysfunction coexist (24, 57). The relative contribution of these pathways depends on lesion type, reperfusion, infection, motility, age, sex, metabolic disease, and treatment. Cell-specific and time-limited perturbations are needed to distinguish direct tissue sensing from peripheral relay mechanisms.

## 5 Temporal and causal interpretation

### 5.1 Time and model are part of the mechanism

The gut-brain interface changes across the stroke course. In the hyperacute phase, sympathetic discharge, intestinal hypoperfusion, epithelial death, altered motility, and reperfusion-related stress may precede detectable community changes. During the early phase, systemic microbial exposure, infection risk, immune-cell activation, and blood–brain-barrier injury can overlap. During recovery, gross permeability may normalize while mucus architecture, community composition, mucosal antibody responses, and metabolite profiles remain abnormal (13–16). A single 24-h or discharge measurement can therefore miss an earlier exposure peak or mistake a recovery-associated signal for an initiating event.

Animal models also differ substantially. Permanent and transient MCAO differ in reperfusion, autonomic stress, injury severity, and mortality; photothrombotic lesions create a different cortical and blood–brain-barrier context. Germ-free status, antibiotic pretreatment, cage effects, diet, fasting, and donor microbiota can dominate microbial measurements. A pig MCAO study showed acute diversity loss with partial recovery by day 5, illustrating why kinetics cannot be transferred directly across species (75). For this reason, experimental claims should report model, occlusion duration, reperfusion status, age, sex, diet, antibiotic exposure, and sampling time whenever available.

The same principle applies to receptor and DAMP studies. Tsukagawa et al. measured serum HMGB1 on admission and day 7 in patients admitted within 24 h of symptom onset (4). Zhang et al. measured serum IFP35 4–6 h after hospital admission and before drug treatment (7). Brea et al. measured peripheral-blood TLR2/TLR4 expression on admission, at 24 h, 72 h and 7 d (12). In the *Enterococcus*/cytolysin study, the clinical cohort was enrolled within 48 h of stroke onset, whereas the mouse infarct endpoint was measured on day 3 after photothrombosis (96). Human biomarker windows and animal intervention windows should therefore be reported separately rather than treated as one continuous timeline.

### 5.2 Host context changes both source and consequence

Age, sex, metabolic disease, baseline microbiota, infection, and organ function influence both signal generation and response. Aged mice show greater intestinal dysfunction, impaired bacterial clearance, and more severe infection-related phenotypes after stroke (21, 22, 67). They also respond differently to SCFAs, valerate, and IL-17 (80, 81, 86). Estradiol-associated epithelial protection and sex-dependent microbiota or immune phenotypes indicate that sex is a biological determinant rather than a nuisance covariate (40, 77–79, 88).

Metabolic disease is another major modifier. Diabetic db/db mice show stronger endotoxemia and may respond to barrier or butyrate-related interventions differently from metabolically healthy animals (57, 85). Type 1 diabetes-associated microbiota changes have been linked to reduced butyrate-producing taxa, microglial myeloid differentiation primary response 88 (MyD88) signaling, and worsened ischemic injury, with partial rescue by butyrate or brain MyD88 manipulation (102). Diabetes-focused mouse-patient comparisons likewise identify context-specific community changes (93). These findings support host-dependent metabolic priming rather than one universal direction of microbial metabolite effect.

In clinical cohorts, dysphagia, aspiration, enteral nutrition, proton-pump inhibitors, bowel medications, antibiotics, urinary or respiratory infection, and renal or hepatic dysfunction can independently alter microbial composition, LPS, and metabolites. These factors are not merely confounders to be adjusted away; they determine whether a source-specific claim is plausible.

### 5.3 What human evidence currently establishes

Human cohorts report intestinal injury markers, time-dependent LPS relationships, loss of anaerobic bacteria, altered SCFAs, oral and gut community signatures, and microbiome features associated with infection, cognition, rehabilitation, recanalization, or functional recovery (25–56, 58). TMAO and related microbial-metabolic pathways have been linked to monocyte, platelet, and vascular phenotypes in stroke and cardiovascular studies (29, 63–65, 89, 90). Mendelian-randomization analysis provides genetic support for associations between selected taxa and outcome but cannot establish the post-stroke tissue source or time window (107). Microbiome-based models can predict stroke-associated pneumonia or functional outcome, but predictive performance does not localize the informative signal to a defined anatomical source or distinguish aspiration- or infection-related exposure (36, 37, 55, 56).

Human studies more strongly support intestinal injury and systemic exposure than anatomical origin. D-lactate and intestinal fatty-acid-binding protein are elevated in acute ischemic stroke, while LPS and related permeability markers show time-dependent associations with severity, infection, and outcome (25–27). These measurements support intestinal injury or systemic exposure, but LPS concentration alone does not identify its source (25–27). Assay and confounder controls are summarized in Section 6.1.

The clinical sampling windows are heterogeneous and should be reported explicitly. Yamashiro et al. enrolled patients within 24 h of symptom onset, collected blood at admission, and obtained the first available stool at a mean of 3.8 ± 2.0 d after admission (28). Yin et al. collected fasting blood within 24 h and the first fecal sample within 48 h of admission (29). Xia et al. enrolled patients within 7 d of onset and collected stool within 48 h of admission (30). Tan et al. enrolled within 48 h of onset and obtained the first spontaneously defecated stool within 72 h of admission; serum measurements were made in the same acute study window (31). Haak et al. collected rectal swabs within 24 h and before antibiotic administration (32), whereas Sun et al. collected feces and fasting blood within 24 h of admission (34). Chang et al. restricted enrolment to patients admitted within 24 h, but did not specify the exact collection interval (33). Henry et al. measured SCFAs at thrombectomy, although the onset-to-sampling interval was not uniform or separately reported (35).

Other human cohorts used later or prognostic windows. Liang et al. enrolled patients within 24 h of onset and defined the rectal prognostic analysis within 24 h of admission, whereas the timing of every oral specimen was not separately stated (36). Lin et al. included patients within 48 h of onset, but the exact admission-to-collection interval was not stated (37). Lledós et al. did not specify an exact onset-to-stool interval (40); Ling et al. collected fresh stool within 1 week of admission (41); Movahedi et al. did not report an exact onset-to-collection interval (42); and Rinawati et al. enrolled patients within 24 h of onset and collected blood and fecal samples within 72 h of admission (43). Ji et al. did not provide a precise onset-to-sampling interval (44). In post-stroke cognitive and rehabilitation studies, Wang et al. used stool collected within 4 d of admission or at 3 months (45), Liu et al. collected feces on admission and assessed cognition at 3 months (46), Hammond et al. sampled at rehabilitation admission, discharge and 3 months without a reported onset-to-rehabilitation interval (47), Chou et al. enrolled patients within 6 h and collected stool before the first meal (48), He et al. collected feces within 24 h after thrombolysis and blood on the first day (49), and Liu et al. collected stool at the end of 4 weeks of robot-assisted gait training while assessing recovery at 90 d (50). For recent cohorts focused on oral-gut overlap, rehabilitation, prediction or outcome (38, 39, 51–53, 55, 56, 94), exact onset-to-sampling intervals were not consistently stated. Across these studies, outcome-assessment time cannot substitute for signal-sampling time, and heterogeneous or unreported onset-to-sampling intervals limit inference about temporal precedence.

The permeability and LPS cohorts also used different windows. Camara-Lemarroy et al. obtained serum during the acute admission within 24 h of onset (25). Błaż et al. measured serum LPS at admission and at 24 h, with a later 3-month assessment (26). Wang et al. sampled plasma at baseline and 24 h after intravenous thrombolysis (27). These measurements support time-dependent exposure associations; causal ordering relative to barrier injury, infection onset, and treatment remains unresolved.

Oral-gut multi-omics introduces another source question. Full-length sequencing across oral plaque and stool identified overlapping genera, altered butyrate-producing taxa, and tryptophan-related metabolic differences in ischemic stroke (39). Such overlap is compatible with exchange between niches, but it does not document movement of a defined organism into blood or brain. Together with the oropharyngeal findings above, it argues that anatomical source should be measured rather than assumed.

**Table 2** summarizes study-level sampling windows, source/route measurements, and the inferences permitted by representative studies.

**Table 2:** Representative primary studies and the inferences they support.

Study	Signal/destination	Model and timing	Source/route approach	Permitted source/route inference and causal-role evidence
Stanley et al. (20)	Viable bacteria; lung and peripheral tissues	Mouse ischemic stroke; permeability preceded dissemination; exact interval NR	16S source analysis, paired tissues, oral inoculation	Source/route inference: links a small-intestinal source to a post-stroke lung community in this model; it does not establish a universal human route, lymph-versus-blood movement, or direct brain delivery. Causal-role evidence: gut-to-periphery dissemination is demonstrated, but a role in secondary brain injury was not tested.
Oyama et al. (23)	Viable bacteria; extra-intestinal organs	Transient MCAO; first 72 h after stroke	Functional permeability, culture/fluorescence *in situ* hybridization (FISH), mucosal immune assessment	Source/route: junctional change without consistent functional leak/translocation. Causal role: not demonstrated in this model.
Kurita et al. (57)	LPS; plasma and ischemic brain	Diabetic db/db mice after transient MCAO; intestinal permeability assessed before and 3 h after MCAO; brain endpoints mainly at 24 h	Barrier assessment, plasma LPS, brain signaling, non-absorbable antibiotic intervention	Source/route: gut-associated endotoxemia in diabetic mice; brain route unresolved. Causal role: polymyxin supports a contribution in this model, not a traced gut-to-brain effect.
Peh et al. (24)	Bacterial-like structures; blood and ischemic brain	Photothrombotic stroke/MCAO; bacterial signals measured at 24 and 72 h, with key quantification at 24 h; germ-free comparison	Immunostaining, absolute PCR, germ-free comparison, sympathetic inhibition	Source/route: microbiota-dependent brain-associated signals; gut-to-brain origin and viability unresolved. Causal role: necessity for injury not established.
Diaz-Marugan et al. (95)	Opportunistic commensals; post-stroke lung	Controlled post-stroke lung-infection model; exact sampling interval NR	Infection and gut-community analyses	Source/route: gut Enterobacteriaceae expansion was not required for lung colonization under the tested conditions. Causal role: the findings did not support a requirement for the gut bloom for this endpoint.
Benakis et al. (70)	Community-dependent immune programming; intestine/meninges	Experimental stroke mice; acute immune-cell endpoints; exact interval NR	Microbiota and gamma-delta T-cell manipulation	Source/route: microbiota-dependent peripheral immune relay; no direct brain entry. Causal role: supported by microbiota/T-cell perturbation in mice.
Huang et al. (58)	Intestinal epithelial TLR4 relay; gut and brain injury	Acute MCAO with recovery cohorts; exact sampling intervals NR	Intestinal epithelial vs brain-specific deletion, pharmacology, rescue	Source/route: epithelial TLR4 relay; ligand and transport route unresolved. Causal role: deletion/pharmacology/rescue support compartment involvement.
Yuan et al. (54)	Acetate-related phenotype; plasma/cortex/recovery	Older adults with stroke plus aged-mouse transfer model; human sampling window NR; mouse recovery/angiogenesis followed longitudinally	Human association plus donor-microbiota transfer and angiogenesis/lymphatic measurements	Source/route: aged-mouse transfer and human acetate association; patient source unresolved. Causal role: community-transfer effect supported; acetate necessity not established.
Yao et al. (39)	Oral and gut communities/metabolites	Human oral-plaque/stool cohort; acute sampling window NR	Full-length 16S sequencing and metabolomics across niches	Source/route: oral/stool overlap; no organismal transfer or systemic source shown. Causal role: association only.
He et al. (38)	Oropharyngeal and gut communities; systemic inflammation	acute ischemic stroke (AIS) with or without preceding infection; collection window NR	Oropharyngeal and gut sequencing plus inflammatory markers	Source/route: oral and gut dysbiosis associate with outcome; systemic source unassigned. Causal role: observational association.
Li et al. (55)	Gut community/coagulation phenotype; outcome	Human AIS prediction cohort; acute study window; exact collection interval NR	Microbiota plus coagulation-based prediction model	Source/route: predictive model, without tissue or route tracing. Causal role: not established.
Chen et al. (101)	Indole-3-propionic acid/TREM2-NLRP3 pathway	Experimental post-stroke model; intervention and pathway endpoints; exact interval NR	Defined microbial/metabolite intervention and pathway analysis	Source/route: defined intervention supports a metabolite pathway in one model; human source/route unresolved. Causal role: experimental contribution, not a universal gut-to-brain mechanism.

Studies are organized according to the primary question they address. The final column distinguishes source/route inference from causal-role evidence and indicates whether causal testing was performed. NR indicates that a precise sampling interval was not reported or separately specified; relative timing is retained where reported in the original study.

### 5.4 Evidence state and causal role are separate

Source tracing can link a detected exposure to its anatomical origin; it does not show that the exposure changed the outcome. A traced signal may be a bystander, a marker of barrier failure, or one contributor among several; causal-role assignment requires temporal precedence and outcome-directed perturbation. Conversely, receptor perturbation can identify biological contribution without resolving anatomical source (20, 24, 57, 58).

### 5.5 Driver, peripheral primer, and amplifier are temporal causal positions

The same intestinal compartment can occupy different causal positions in different models and time windows. We use driver for a gut-derived signal that is temporally upstream and necessary or sufficient for a defined secondary injury. We use peripheral primer for a gut-associated exposure that changes myeloid, endothelial, or other peripheral state and thereby modifies the later response to a brain lesion without requiring direct microbial sensing in brain tissue. We use amplifier for a later process that increases the magnitude of an inflammatory cascade that has already been initiated.

The experimental criteria for assigning these roles are outlined in Section 6.2.

## 6 A source-aware evidence package and translational implications

### 6.1 Minimum evidence package for source and route attribution

Operationally, source claims require a defined signal, source-discriminating linkage, matched recovery in the target compartment, and controls for plausible competing sources; route claims add pathway-matched measurements (20, 23, 24, 95). Paired gut-target detection or a germ-free comparison alone supports concordance or microbiota dependence, not intestinal source attribution (20, 24). Receptor engagement or sensing-cell specificity establishes recognition rather than anatomical origin or transport route (11, 57, 59).

Assays should be matched to both the claim and signal class. Functional permeability probes test epithelial passage; tight-junction, mucus, epithelial-death, and mucosal-immune measures describe barrier state but do not by themselves demonstrate systemic transport (14–17, 23). For LPS, interpreting concentration alongside LBP, soluble CD14, neutralization or recovery controls, infection adjudication, and renal/hepatic data strengthens exposure assessment but does not establish intestinal origin or crossing route (25–27, 57, 59). For bacterial DNA or structures, negative controls and spatial localization address contamination and tissue position, whereas culture or a validated viability assay establishes whether living bacteria are present (20, 23, 24, 95). Targeted chemistry establishes metabolite identity and concentration; portal/systemic sampling, isotope tracing, or defined-source colonization can test intestinal production or transfer, while diet, medication, and host clearance remain alternative determinants (54, 60–65, 80–92, 97–104).

**Figure 2** summarizes the evidence packages for source attribution, route inference, and causal-role assignment.

**Figure 2:**
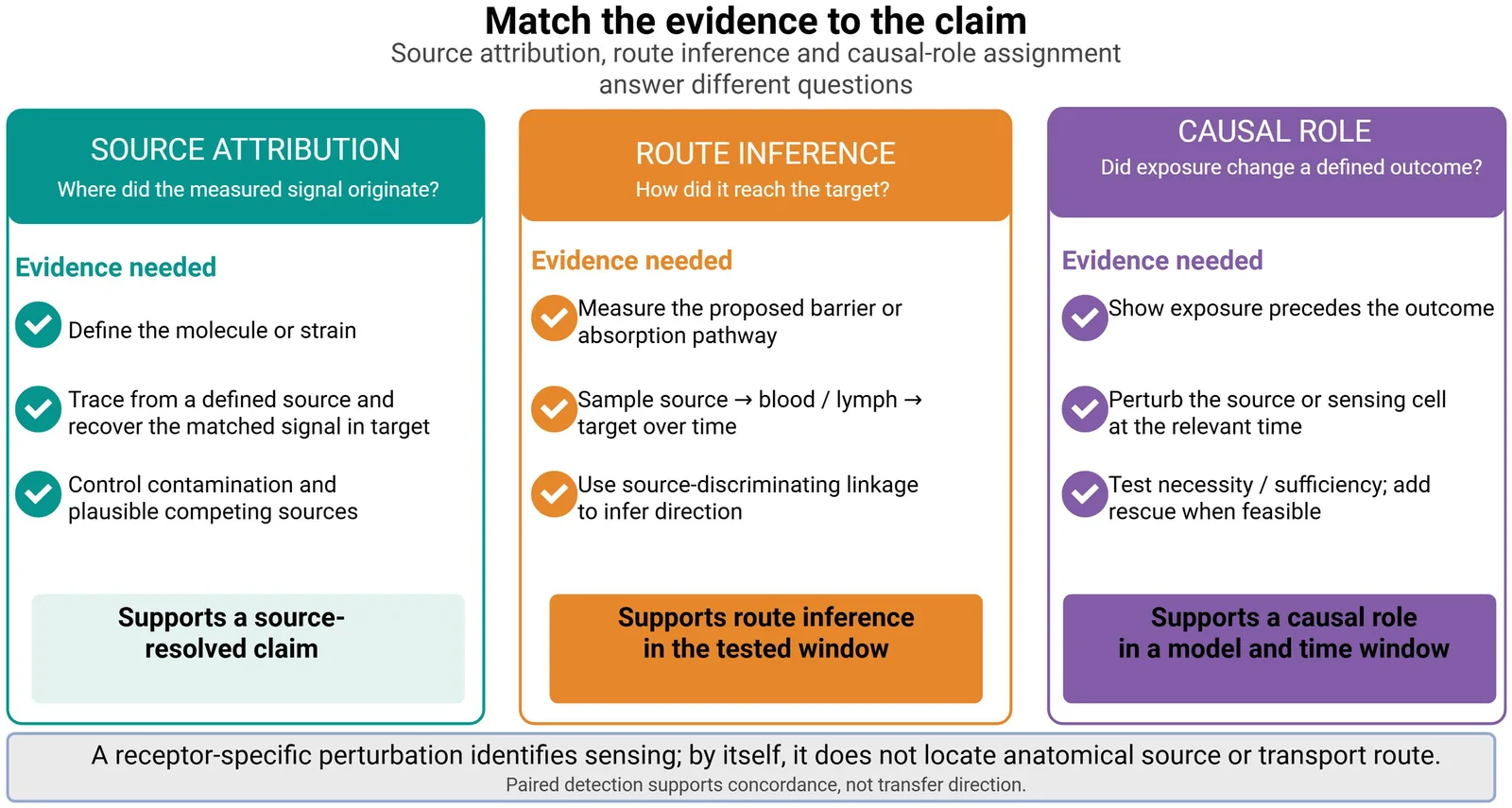
Evidence required for source attribution, route inference, and causal-role assignment. Source attribution requires a defined signal and source-discriminating linkage to a matched target; route inference adds measurements matched to the proposed pathway; causal-role assignment requires exposure to precede the outcome and appropriately timed source- or cell-specific perturbation, with rescue where feasible. Receptor perturbation identifies sensing but does not establish anatomical origin or transport route.

### 6.2 Additional evidence required for causal assignment

Causal assignment asks whether a defined exposure changes a prespecified tissue or functional outcome. The exposure should be measured before that outcome, and a source- or cell-specific perturbation should be applied within the relevant time window; necessity or sufficiency tests and rescue, where feasible, strengthen the inference (20, 57, 58, 66, 70). Source-directed manipulation tests whether a source contributes, whereas cell-specific manipulation identifies the responding compartment; receptor perturbation alone cannot determine the ligand’s anatomical origin (3–12, 57, 58, 66, 70, 96, 101).

Broad or prolonged receptor deletion can alter antimicrobial defense, tissue repair, microbial ecology, or bacterial clearance. Antibiotic, probiotic, and fecal-transfer studies test microbiota dependence; source tracing is needed to assign anatomical origin, and outcome-directed perturbation is needed to establish causal role (57, 58, 66, 70, 96, 101).

### 6.3 Barrier and autonomic protection

Barrier and autonomic interventions test whether post-stroke intestinal physiology can be modified. Studies manipulating sympathetic or vagal output, epithelial integrity, motility, mucosal defense, or epithelial TLR5/TLR4 signaling provide proof-of-concept for modifiable checkpoints (13, 14, 17, 58, 87, 108). To test whether a barrier intervention reduces dissemination, barrier measurements should be paired with source-to-target exposure measurements (20, 23, 24, 95).

These interventions may also affect blood pressure, cerebral perfusion, intestinal transit, and susceptibility to infection. Studies should report the treatment window alongside neurological and infection outcomes, because an intervention that helps during early epithelial injury may have different effects during later antimicrobial defense or tissue repair (1–17, 87, 108).

### 6.4 Microbiota- and metabolite-directed interventions

Microbiota- and metabolite-directed interventions test whether selected communities or molecules modify stroke phenotypes, whereas endogenous source and route require separate measurements (24, 54, 69–71, 81–86, 97–104, 109–112). More source-informative examples target defined candidates: *Duncaniella muris*-associated restoration of indole-3-propionic acid was linked to reduced TREM2-dependent NLRP3 pyroptosis (101), and *Akkermansia muciniphila* and indole-lactic-acid-related signaling were associated with barrier and ferroptosis-related protection (103, 104).

Acetate-associated recovery findings and young-donor microbiota transfer in aged mice support context-specific recovery hypotheses (54, 109). Diabetes-associated transfer studies separately underscore the influence of host metabolic state (102, 112). Outcomes should be interpreted in light of dose, chemical species, host age, metabolic disease, diet, renal function, and sampling time; SCFAs, TMAO, hydrogen sulfide, indole derivatives, and branched-chain amino-acid pathways are context-dependent and are not universal barrier biomarkers (60–65, 80–92, 97, 98).

### 6.5 Selective innate-immune modulation and safety

Selective innate-immune modulation tests which receptor or immune compartment contributes to a defined outcome once the candidate signal and route are measured. Intestinal epithelial TLR4 and peripheral immune-relay studies illustrate how selective perturbations can localize the sensing or relay compartment (58, 66, 70).

Innate pathways also support antimicrobial defense and tissue repair, so broad or prolonged blockade may compromise host defense and obscure a time-specific mechanism. Studies should report target engagement and intervention timing alongside infection and neurological outcomes (1–12, 58, 66, 96). No intestinal-origin biomarker threshold or source-specific therapeutic window has yet been clinically validated (25–27, 36, 37, 55, 56, 95).

### 6.6 Negative and context-dependent results should shape translation

Negative and context-dependent results help define the limits of translation. Across these models, upstream changes did not consistently predict downstream outcomes: functional permeability or bacterial translocation was not consistently detected, gut-bloom suppression did not prevent lung colonization, and butyrate altered microglial responses without improving behavior (23, 95, 113).

Intervention studies should therefore prespecify which link they test—barrier modification, reduced signal in blood or target tissue, receptor/sensing-cell response, or tissue/behavioral outcome—and measure relevant neurological and infection-related safety outcomes. Until anatomical source, route, biomarker threshold, therapeutic window, and clinical benefit are prospectively validated, biomarker-guided treatment remains a research hypothesis (20, 23–27, 36, 37, 55–58, 95).

## 7 Conclusion

Ischemic stroke engages a well-established brain-derived DAMP response, while autonomic and intestinal changes can alter microbial ecology, mucosal defense, metabolite exposure, and peripheral immune state. Experimental studies report model-dependent intestinal injury, source-linked gut-to-peripheral dissemination in selected settings, and microbiota-associated signals in ischemic brain tissue (20, 24). Human cohorts primarily contribute association, exposure, and prediction evidence across heterogeneous sampling windows (25–56, 58, 107). Together, current findings support context-dependent intestinal involvement after ischemic stroke, but they have not yet connected a source-defined intestinal signal, a measured brain-directed route, and a secondary brain consequence in one causal sequence.

Future studies should pair source-specific tracing and longitudinal compartment sampling with appropriately timed source- or cell-specific perturbation and rescue where feasible. This design can determine whether gut-related signals function as exposure markers, peripheral primers, drivers, or amplifiers in defined models and time windows.
